# Impact of Long-Term Cryopreservation on Blood Immune Cell Markers in Myalgic Encephalomyelitis/Chronic Fatigue Syndrome: Implications for Biomarker Discovery

**DOI:** 10.3389/fimmu.2020.582330

**Published:** 2020-11-17

**Authors:** Elisabet Gómez-Mora, Jorge Carrillo, Víctor Urrea, Josepa Rigau, José Alegre, Cecilia Cabrera, Elisa Oltra, Jesús Castro-Marrero, Julià Blanco

**Affiliations:** ^1^IrsiCaixa AIDS Research Institute, Institut d’Investigació en Ciències de la Salut Germans Trias i Pujol, Badalona, Spain; ^2^Rigau Private Clinic, Tarragona, Spain; ^3^Division of Rheumatology, ME/CFS Clinical Unit, Vall d’Hebron University Hospital, Universitat Autònoma de Barcelona, Barcelona, Spain; ^4^School of Medicine, Universidad Católica de Valencia San Vicente Mártir, Valencia, Spain; ^5^Division of Rheumatology, ME/CFS Research Unit, Vall d’Hebron Hospital Research Institute, Universitat Autònoma de Barcelona, Barcelona, Spain; ^6^Chair in Infectious Diseases and Immunity, Centre for Health and Social Care Research (CESS), Faculty of Medicine, University of Vic, Central University of Catalonia (UVic–UCC), Vic, Spain

**Keywords:** cryopreservation, immunophenotyping, immune biomarkers, myalgic encephalomyelitis, chronic fatigue syndrome, freeze-thaw process

## Abstract

Myalgic Encephalomyelitis/Chronic Fatigue Syndrome (ME/CFS) is a complex neuroimmune disorder characterized by numerous symptoms of unknown etiology. The ME/CFS immune markers reported so far have failed to generate a clinical consensus, perhaps partly due to the limitations of biospecimen biobanking. To address this issue, we performed a comparative analysis of the impact of long-term biobanking on previously identified immune markers and also explored additional potential immune markers linked to infection in ME/CFS. A correlation analysis of marker cryostability across immune cell subsets based on flow cytometry immunophenotyping of fresh blood and frozen PBMC samples collected from individuals with ME/CFS (n = 18) and matched healthy controls (n = 18) was performed. The functionality of biobanked samples was assessed on the basis of cytokine production assay after stimulation of frozen PBMCs. T cell markers defining Treg subsets and the expression of surface glycoprotein CD56 in T cells and the frequency of the effector CD8 T cells, together with CD57 expression in NK cells, appeared unaltered by biobanking. By contrast, NK cell markers CD25 and CD69 were notably increased, and NKp46 expression markedly reduced, by long-term cryopreservation and thawing. Further exploration of Treg and NK cell subsets failed to identify significant differences between ME/CFS patients and healthy controls in terms of biobanked PBMCs. Our findings show that some of the previously identified immune markers in T and NK cell subsets become unstable after cell biobanking, thus limiting their use in further immunophenotyping studies for ME/CFS. These data are potentially relevant for future multisite intervention studies and cooperative projects for biomarker discovery using ME/CFS biobanked samples. Further studies are needed to develop novel tools for the assessment of biomarker stability in cryopreserved immune cells from people with ME/CFS.

## Introduction

Myalgic Encephalomyelitis (ME), also known as Chronic Fatigue Syndrome (CFS), is a multisystem, complex and extremely debilitating chronic illness. The 2015 Institute of Medicine (IOM) committee report estimated that ME/CFS could potentially affect up to 2.5 million people in the US, and as many as 17-24 million worldwide, of whom roughly 90% are undiagnosed (1). There is no proven specific cause, no accurate and objective diagnostic test, and no universally effective treatment for ME/CFS. Beyond the puzzling post-exertional malaise as the cardinal symptom that cannot be alleviated by rest, ME/CFS patients also present a plethora of unspecific symptoms including debilitating fatigue, unrefreshing sleep, gastrointestinal problems, orthostatic intolerance, cognitive impairments, and pain/myalgia that change in frequency and severity over time and differ from patient to patient (2).

Despite exhaustive research in the past decades, the pathogenesis of ME/CFS is not yet completely understood; prognostic indicators, efficient targeted treatments or coping clinical approaches remain elusive. It has been hypothesized that ME/CFS is a multifactorial condition in which a combination of immunogenetic and shared environmental factors coexist (3). The immunological basis of the disease is highlighted by evidence linking it to autoimmunity, and by the potential role of pathogens as triggers (4–6), leading to the general belief that some disrupted immunometabolic and neuroinflammatory pathways may be associated with ME/CFS (7–9).

Currently, there is no single accurate ‘gold standard’ biomarker for diagnosis of ME/CFS. Several immune mediators, such as circulating cytokines/chemokines or immune cell subset markers, have been investigated as candidate biomarkers for the disease. These biomarkers may become valuable tools for the diagnosis, prognosis and response to treatment in ME/CFS sufferers (10). To this end, our group examined the phenotype and function of NK, B and T cell subsets in 22 Spanish ME/CFS patients, concluding that the cases showed an unaffected B-cell compartment and an altered T and NK cell phenotype. Higher expression of NKp46 and CD69 markers and lower expression of CD25 in NK cells were evidenced, along with an apparently poor response of the T-cell compartment, with increased numbers of regulatory T cells (Tregs) and lower activation and frequency of effector memory CD8^+^ T cells. Furthermore, particular combinations of these disease-biased parameters identified eight robust immune markers that were able to discriminate between ME/CFS patients and healthy controls with a sensitivity of 100% and specificity of 79% based on ROC analysis (7).

Biomarker validation studies involve large numbers of biospecimens that could be effectively supplied by biobanks. This key service platform facilitates worldwide network collaboration by harmonizing clinical data and by providing high-quality standardized biosamples, which are obtained, processed, and preserved through optimized good manufacturing practice methods. Specifically, in the field of ME/CFS biomedical research, biobanking facilities such as SolveCFS (https://solvecfs.org) in the US and the ME/CFS Biobank (https://cureme.lshtm.ac.uk) in the UK (11, 12), already have large biospecimen collections and associated anonymized clinical information available for future biomedical research, including biomarker discovery. However, biobanking involves storage, often long-term (>6 months), and downstream freeze/thaw processes, which may potentially explain the alterations in sensitive markers. Since our initial study was performed in fresh samples, it seemed necessary to confirm the cryostability of the discriminant immune markers identified before proceeding with population validation. No previous studies have explored the effects of storage time of blood samples on the cryostability of immune markers in ME/CFS, and to the best of our knowledge, no studies have attempted to establish standard operating procedures on ME/CFS-focused sample quality control.

The aim of this study was to assess the impact of long-term biobanking on our previously identified immune markers in peripheral blood mononuclear cell (PBMC) subsets of individuals with ME/CFS and healthy controls and to explore additional immune markers in frozen Treg and NK cells from the same previously studied cohort. Ideally, study biomarkers unaffected by sample cryopreservation would be identified.

## Materials and Methods

### Ethics Statement

This study was carried out in accordance with the recommendations of the local Research Ethics Committees. All subjects provided written signed informed consent prior to participation in accordance with the Declaration of Helsinki. The study protocol was approved by the local Health Research Ethics Board (IrsiCaixa, IGTP Committee, Badalona, Spain; reference number: EO-10-007).

### Study Setting and Participants

In this retrospective, cross-sectional, case-control cohort study, 22 potentially eligible ME/CFS patients who met the 1994 CDC/Fukuda definition (13) and recruited from two cohorts of ME/CFS outpatient clinics (Clinics Omega Zeta, Tarragona, Spain and Vall d’Hebron University Hospital, Barcelona, Spain) were initially evaluated by two physicians specialized in ME/CFS (JR and JA) as part of our initial study on immune biomarker discovery in 2013 (7). The 18 PBMC samples analyzed from each cohort were selected after three years of cryopreservation on the basis of cell availability for the programmed assays. All routine laboratory testing yielded normal results (*data not shown*). As previously reported, fatigue severity was assessed through the self-administered 40-item Fatigue Impact Scale (FIS-40) validated in the Spanish ME/CFS population (14, 15). Unfortunately, no cells were available from the same healthy controls of the original 2013 study. Therefore, eighteen age- and sex-matched healthy controls from the local community recruited through word-of-mouth and advertisements and having cryopreserved PBMC samples (≥ 3 years) were selected. All participants were of Caucasian descent, from the same geographical area, and had a sedentary lifestyle at the time of study. **Table 1** shows the sociodemographic and clinical characteristics of the study participants. Exclusion criteria were acute infectious disorders in the previous 4 weeks, past/present neurological, metabolic or infectious disorders, immune disorders such as IgA deficiency, psychosis, major depression, cardiac, autoimmune and hematological disorders, allergy-related diseases, dermal or chronic inflammation disorders; medical conditions that required glucocorticoid, statin, or antiviral drug use; pregnancy/breastfeeding; or any symptom/signs that might be confused with those of ME/CFS.

**Table 1:** Demographic and clinical characteristics of the study population at baseline.

Variables	ME/CFS (n = 18)	HCs (n = 18)
Age (years)	50 (45–57)	48 (38–59)
Sex (female/male)	13/5	13/5
Illness duration at diagnosis (years)	3.2 ± 7.2	–
Fatigue severity	3 (2–3)	–
**Triggers**		
Infectious	63	–
Non-infectious	37	–
**Comorbid conditions**		
Anxiety/depression	47	–
Myalgia/tendinopathy	36	–
Multiple Chemical Sensitivity	14	–
**Medications**		
Analgesics/NSAIDs	33	–
Antidepressants/anxiolytics	44	–
Homeopathy	44	–
Antioxidant supplements	44	–

Data are expressed as median ± standard deviation (SD) for continuous variables, and as number of cases (%) for categorical variables, except for age and fatigue severity (median, interquartile range). NSAIDs, non-steroidal anti-inflammatory drugs.

### Blood Collection and Processing

Peripheral blood (25 ml) was collected by venipuncture directly into EDTA-containing tubes (K2E Vacutainer, BD) from each participant. An aliquot was used for immediate immunophenotyping on the day of blood collection; remaining whole blood was processed for PBMC isolation by density gradient centrifugation using Ficoll^®^ Paque Plus (GE Healthcare, catalog no. 17-1440-02). Isolated PBMCs were washed and resuspended in a standard cryomedium containing 90% fetal bovine serum (FBS, Gibco catalog no. 10091148) plus 10% dimethyl sulfoxide (DMSO, Sigma-Aldrich, catalog no. D2650). One milliliters aliquots with 1x10^7^ cells/ml were gradually frozen in Mr. Frosty device at −80°C for at least 48 h and then stored into a cryo-tank containing liquid nitrogen at −196°C until use.

### Cell Viability and Staining Assay

Cryopreserved PBMC-containing vials were rapidly thawed into pre-warmed bath at 37°C for 5 min and washed with PBS, and 1x10^6^ cells were stained with eFluor™ 506 dye (200 μg/ml, ThermoFisher catalog no. 65-0866-18) to analyze cell viability using flow cytometry analysis. Cellular yield was calculated as number of living cells/ml. Viability was defined as % of living cells from total cell count. Cells negative for eFluor™ 506 stains were regarded as viable. PBMCs were then washed with PBS and stained with different combinations of fluorochrome-conjugated anti-human antibodies. Besides the antibodies previously used to compare expression between fresh blood and frozen PBMCs (7), four new combinations of antibodies were added in order to explore new immune markers in the study (**Table 2**).

**Table 2:** Panel description of fluorochrome-conjugated monoclonal antibodies used in this study for staining by multicolor flow cytometry.

	Panel 1	Panel 2	Panel 3	Panel 4
Fluorochromes	Treg cells	T cell function	Effector T cells	NK cells
eFluor™ 506	Viability	Viability	Viability	Viability
FITC	Ki67	**IFN-γ**	–	–
PE	FOXP3	FOXP3	CD28	**NKG2C**
PE-Cy™7	–	**IL-4**	–	CD56
PerCP-Cy™5.5	CD3*	CD3*	CD4	CD69*
APC	–	–	CD27	CD57
APC-H7	CD4*	CD4*	CD3	CD16
Alexa Fluor^®^ 700	CD8*	CD8*	–	–
PE-CF594	CD25*	CD25*	–	CD25*
Alexa Fluor^®^ 647	CD127	CD127	–	–
PE-Cy™7	**CD45RA**	–	–	–
V450	–	–	–	NKp46
V500	–	–	CD8	–
BV421	**CD73**	**TGF-β1**	–	–
BV510	–	–	–	**CD3/CD19**
BV650	**CD39**	–	–	–
BV786	–	**IL-17**	–	–

•Asterisks denote a change in the fluorochromes.

•Antibodies highlighted in bold denote new immune markers added in this study.

### T Effector Cell Immunophenotyping

PBMCs (0.5 million) were incubated for 15 min at room temperature with the anti-CD3-APC-Cy™7 (clone SP34-2), anti-CD4-PerCP (clone L78SK3), anti-CD8-V500 (clone SK1), anti-CD27-APC (clone M-T271) and anti-CD28-PE (clone CD28.2) monoclonal antibodies (all from BD Biosciences) according to standard protocols. Cells were then washed with PBS, fixed with 1% formaldehyde in PBS (Sigma-Aldrich, catalog no. 1004960700) for 15 min at room temperature and analyzed by flow cytometry.

### Regulatory T Cell Immunophenotyping

To determine the Treg profile and function, 1x10^6^ PBMCs were incubated for 15 min at room temperature with the anti-CD3-PerCP-Cy™5.5 (clone UCHT1), anti-CD4-APC-H7 (clone SK3), anti-CD8-Alexa Fluor^®^ 700 (clone RPA-T8), anti-CD25-PE-CF594 (clone M-A251), anti-CD127-Alexa Fluor^®^ 647 (clone HIL-7R-M21), anti-CD45RA-PE-Cy™7 (clone HI100), anti-CD39-BV650 (clone TU66), and anti-CD73-BV421 (clone AD2) monoclonal antibodies (all from BD Biosciences). Cells were then washed and fixed/permeabilized (eBioscience, catalog no. 00-5521-00) using FOXP3 staining buffer (eBioscience, catalog no. 00-5523-00), and finally stained with the combination of conjugated antibodies anti-Ki67-FITC (BD Biosciences, catalog no. 556026) and anti-FOXP3-PE (BD Biosciences, clone 259D/C7) for nuclear epitope staining. Next, cells were washed with PBS, fixed with 1% formaldehyde in PBS (Sigma-Aldrich, catalog no. 1004960700) for 15 min at room temperature and analyzed by flow cytometry.

### NK Cell Immunophenotyping

For NK characterization, 2x10^6^ PBMCs were incubated for 15 min at room temperature with the following conjugated antibodies: anti-CD3-BV510 (clone UCHT1), anti-CD19-BV510 (clone SJ25C1), anti-CD16-APC-Cy™7 (clone 3G8), anti-CD56-PE-Cy™7 (clone B159), anti-NKp46-V450 (clone 9E2), anti-NKG2C-PE (clone 134591), anti-CD69-PerCP (clone L78), anti-CD57-APC (clone NK-1), and anti-CD25-PE-CF594 (clone M-A251) monoclonal antibodies (all from BD Biosciences). Cells were then washed with PBS, fixed with 1% formaldehyde in PBS (Sigma-Aldrich, catalog no. 1004960700) for 15 min at room temperature, and analyzed by flow cytometry.

### Intracellular Cytokine Staining Assay

Briefly, 2 × 10^6^ PBMCs per condition were thawed, washed and placed in culture at 4 million cells/ml in RPMI-1640 medium (Gibco by Life Technologies, catalog no. 11875-093) supplemented with penicillin/streptomycin (Gibco by Life Technologies, catalog no. 15140122) and 10% FBS (Seradigm, catalog no. 1500-500). After 2 h, cells were stimulated “*in vitro”* with PMA (62.5 ng/ml, Sigma-Aldrich, catalog no. P1585) and ionomycin (0.6 µM, Sigma-Aldrich, catalog no. I9657) to induce cytokine production in the presence of brefeldin A (10 μg/ml, BD Biosciences, catalog no. 555029) and monensin (2 μM, BD Biosciences, catalog no. 554724) and incubated for 5 h at 37°C as described (16). Cells were then stained for 15 min with anti-CD3-PerCP-Cy™5.5 (clone UCHT1), anti-CD4-APC-H7 (clone RPA-T4), anti-CD8-Alexa Fluor^®^ 700 (clone RPA-T8), anti-CD25-PE-CF594 (clone M-A251), and anti-CD127-Alexa Fluor^®^ 647 (clone HIL-7R-M21) conjugated antibodies (all from BD Bioscience), washed and fixed/permeabilized (eBioscience, catalog no. 88-8824-00) using FOXP3 staining buffer (eBioscience, catalog no. 00-5523-00), and finally stained with the following intracellular monoclonal antibodies: anti-IFN-γ FITC (clone B27), anti-IL-17A-BV786 (clone N49-653), anti-IL-4-PE-Cy™7 (clone 8D4-8), and anti-TGF-β1-BV421 (clone TW4-9E7) (all from BD Biosciences). At this point, cells were washed twice with PBS and fixed with PBS containing 1% formaldehyde (Sigma-Aldrich, catalog no. 1004960700). As negative control, unstimulated cells were included in each experiment. All stained samples were acquired on an LSRFortessa flow cytometer using a plate HTS loader (BD Biosciences), except for T effector cell immunophenotyping (LSR-II flow cytometer, BD Biosciences). Data analysis was performed using FlowJo LLC software v10.4.2 (Tree Star, Ashland, OR, USA). A minimum of 10,000 total events were recorded for each panel and condition. Although most antibodies were maintained from our original study, the addition of new markers (highlighted in bold on **Table 2**) and the changes in configuration of the flow cytometer resulted in fluorochrome changes in several markers (marked by asterisks on **Table 2**). We tried to minimize the impact of these changes by restricting them to highly expressed molecules (i.e., CD3, CD4 or CD8).

### Statistical Analysis

Continuous variables were expressed as medians ± IQR (interquartile range). Qualitative variables were expressed as percentages. Descriptive statistics and data visualization (graphs) were generated using GraphPad Prism version 7.0 (GraphPad Software Inc., San Diego, USA). Group comparisons were performed by either Chi-square test for continuous variables or the Fisher’s exact test for categorical variables. Differences between quantitative variables were compared using the non-parametric Mann-Whitney *U* test or χ^2^ test, as appropriate. Comparisons between fresh and thawed samples were assessed in paired data using the Wilcoxon signed-rank test (two-tailed). Correlation analysis between continuous variables was calculated using the non-parametric Spearman rank test to explore the nature of the relationship between two continuous variables and multiple testing and further adjusted by the false discovery rate. The comparison of slope values with a full identity (slope = 1) was performed after linear regression analysis using the F-test. All statistical analyses were performed using SPSS v23.0 (SPSS, Chicago, IL, USA). All experiments included a minimum of three independent replicates. Statistical significance was established at a *p*-value ≤ 0.05. All *p*-values are documented in the corresponding figures and legends.

## Results

### Population Characteristics

ME/CFS participants were selected according to sample availability from the previously defined cohort (7). Control individuals were selected from biobanked samples and matched by age and gender; the overlap with the former control group was minimal, which precluded direct comparison of fresh and cryopreserved samples in this group. As shown in **Table 1**, no differences in age or gender were observed among participants. Most participants reported infection as the disease trigger (63%). Additional factors that may have altered the immune function in ME/CFS (comorbidity and polypharmacy) were also recorded. Among comorbidities, anxiety/depression was the most prevalent (47%), although myalgia/tendinopathy (36%) and self-reported multiple chemical sensitivity (14%) also appeared frequently. The main potential interferences of self-reported poly-drugs in the ME/CFS cohort studied were homeopathy (44%), antioxidant supplements (44%), NSAIDs (33%, mainly paracetamol), and antidepressants/anxiolytics (44%), which were evenly distributed across participants.

### Validation of Previously Described Markers

To evaluate the stability after cryopreservation of the ME/CFS immune markers identified in our previous study and thus to determine the possibility of applying our diagnostic criteria to biobanked ME/CFS collections with a view to population validation, we selected the most prominent subpopulation frequencies and immune markers found to be altered in fresh ME/CFS blood samples. We reassessed them on thawed replica aliquots using a similar methodology to that applied in our original 2013 study (7), after a three-year interval. The cohorts consisted of 18 patients with ME/CFS and 18 matched healthy controls, since aliquots from four of the original ME/CFS cases and 12 healthy controls were no longer available. **Table 2** shows the panel of fluorochrome-conjugated monoclonal antibodies used in the immunophenotypic characterization of frozen PBMCs. For the previously identified T cell biomarkers (7), we measured the frequency of the Treg cells CD3^+^CD4^+^CD25^bright^FOXP3^+^CD127^-^ (natural Tregs) using the antibodies listed as panel 1 and quantified them as % of CD4^+^ T cells. The same panel also allowed for the quantification of the percentage of CD4^+^ T cells expressing the proliferation marker Ki67. The intracellular cytokine staining assay was performed using panel 2 antibodies. In addition, effector CD8^+^ T cells were quantified as CD3^+^CD8^+^CD28^+^CD27^-^ using panel 3 antibody combinations as listed, and the frequency of CD56^+^CD3^+^ T cells was assayed with panel 4 antibodies. The latter panel was also used to analyze NK cell subpopulations, which were defined as CD3^-^CD19^-^CD56^+^CD16^+^, which the expression of the potential ME/CFS markers CD25, CD69, NKp46 and CD57 was analyzed.

### Analysis of T Cell Markers in Fresh and Frozen Samples

**Figure 1:**
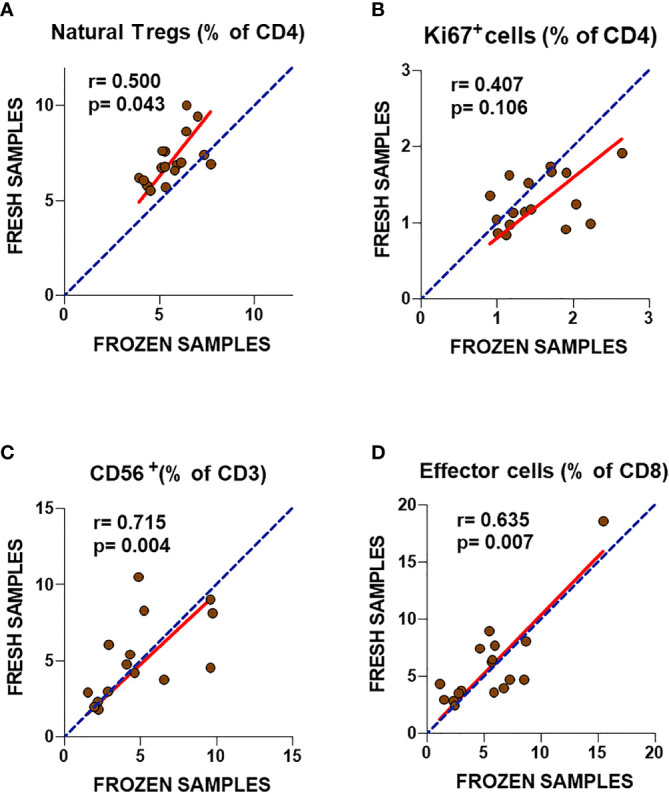
Analysis of T cell subset markers in fresh and frozen samples from ME/CFS patients **(A–D)**. Frequencies of the analyzed T cell subsets in fresh (2013) and frozen (2016) PBMC samples from the same participants are shown. Complete identity between fresh and frozen cells is illustrated by blue dotted lines. Linear regression of data is illustrated by red lines. Spearman correlation coefficients and *P*-values are shown for each panel.

Comparison of ME/CFS T cell markers in fresh blood and cryopreserved PBMCs from ME/CFS participants is shown in **Figure 1**. The percentage of natural Treg cells (CD3^+^CD4^+^FOXP3^bright^CD25^+^CD127^-^) showed significant correlations (r = 0.50; p = 0.043) between frozen and fresh cells, with a trend towards decreased frequencies in frozen samples (**Figure 1A**). Similarly, significant correlations were obtained for classic Treg cells (CD3^+^CD4^+^FOXP3^bright^CD25^+^; r = 0.54, p = 0.032) (*data not shown*). A non-significant correlation (r = 0.407; p = 0.106) was observed for the frequency of CD4^+^Ki67^+^ cells which tended to show higher values in frozen samples compared to fresh blood data (**Figure 1B**). By contrast, CD56 expression in T cells (NKT cells) and the frequency of effector CD8^+^ T cells showed better correlation between fresh and frozen samples (r = 0.715; p = 0.004 and r = 0.635; p = 0.007, respectively), with linear regressions close to identity values (slope closer to 1) (**Figures 1C, D**). These results indicate the robust reproducibility, and thus cryostability, of some markers assayed during long-term storage periods, with the exception of Ki67. However, despite the significant correlations, the slopes of linear regression analysis indicated significant differences from identity (slope = 1) for natural Tregs and Ki67+ CD4 T cells (p = 0.0008 and p = 0.0035, respectively, F-test). Slope value > 1 was found for natural Tregs indicating weaker expression in frozen compared to fresh cells, while the slope value < 1 for Ki67+ cells, indicated the opposite. These changes were confirmed by comparing data obtained from fresh and frozen samples using the Wilcoxon paired test, which showed significantly lower values for the frequency of natural Tregs (p = 0.0002) and significantly higher values for Ki67+ CD4 T cells (p = 0.0461) in frozen samples (**Supplementary Figure 1A**, upper panel). However, no statistically significant differences were shown for the frequency of CD56+ cells (p = 0.5995) and effector CD8 T cells (p = 0.3289) (**Supplementary Figure 1A**, lower panel). Despite the potential impact of cryopreservation, our data suggest that the use of biobanked PBMCs to validate previously identified immune markers may be feasible.

**Figure 2:**
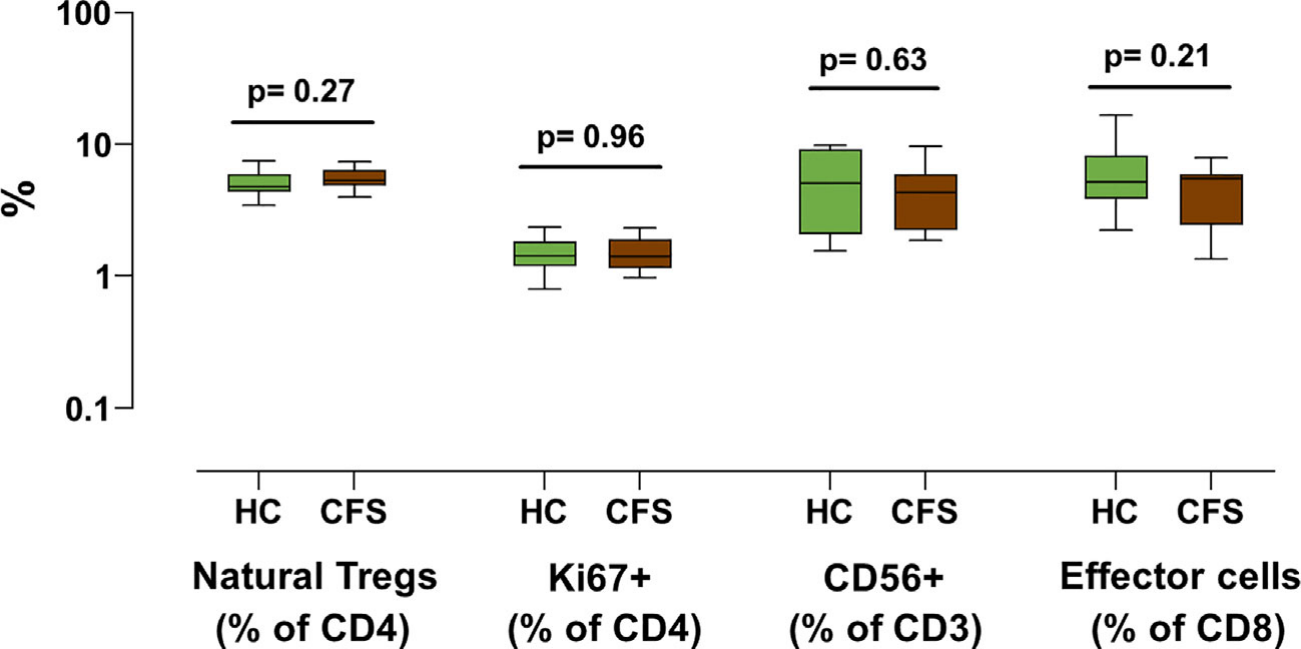
Analysis of T cell subset markers in frozen PBMC samples from individuals with ME/CFS and healthy controls. The indicated T cell subsets were assessed in frozen PBMC samples from 18 ME/CFS individuals (brown) and 18 healthy controls (green). Data is shown as median values with interquartile range (boxes), plus minimal and maximal observations (bars). *P*-values are indicated for each set of groups compared.

To further confirm this possibility, frozen PBMC samples from ME/CFS cases and healthy controls were compared. Unexpectedly, none of the markers that had shown significant differences in our previous study performed in 2013 presented statistically significant differences across groups (**Figure 2**). Although ME/CFS patients showed a trend towards lower levels of effector CD8^+^ T cells and CD56^+^ T cells and higher levels of natural Treg cells, consistent with the differences found in our 2013 study, on this occasion, no significant differences could be established between groups. The fact that two of the immune cell subsets with lower p-values (Tregs and effector T cells, p = 0.043 and p = 0.007; respectively) presented significant correlations with fresh blood values (**Figures 1A, D**) argues in favor of pursuing the evaluation of these cell subsets of larger sets of ME/CFS biobanked samples, in preference to the other two T cell subpopulations assayed (CD3^+^CD56^+^ and CD4^+^Ki67^+^) (**Figure 2**).

### Analysis of NK Cell Surface Markers in Fresh and Frozen Samples

**Figure 3:**
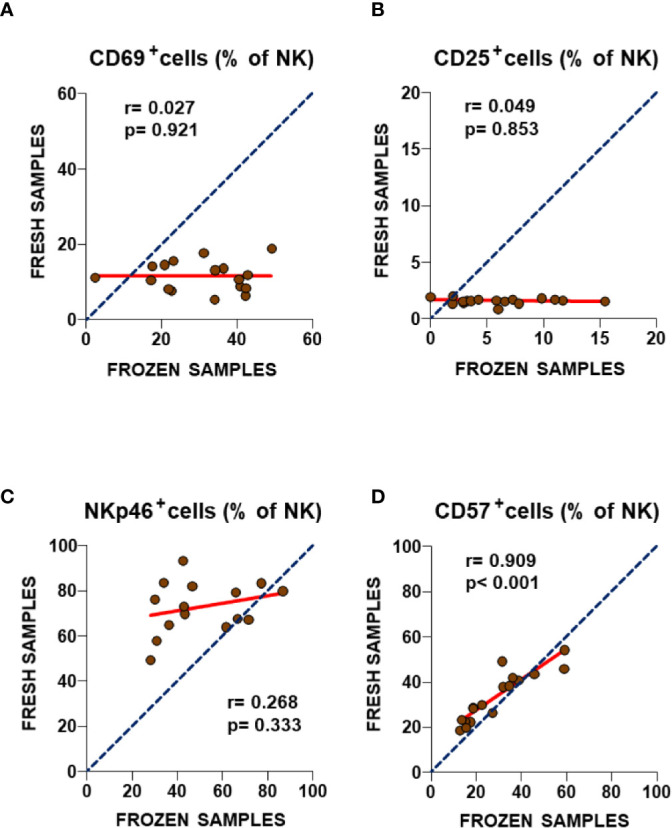
Analysis of NK cell subset markers in fresh and frozen samples from ME/CFS patients **(A–D)**. Frequencies of the analyzed NK cell subsets in fresh (2013) and frozen (2016) blood samples from the same participants are shown. As in **Figure 1**, complete identity between fresh and frozen cells is illustrated by blue dotted lines. Linear regression of data is illustrated by red lines. Spearman correlation coefficients and *P*-values are shown for each panel.

A similar cryostability analysis was performed of four additional NK cell immune markers after long-term storage and freeze/thawing process (**Figure 3**). This time, particularly poor correlations were observed for both CD69 and CD25 between fresh and frozen samples (**Figures 3A, B**) with slope values significantly lower than one (p< 0.0001 for both, F-test), indicating increased staining for both markers after the thawing process (p = 0.0002 and p = 0.0003 respectively, Wilcoxon paired test, **Supplementary Figure 1B**, upper panel). A similarly poor correlation was also observed for the expression of the NKp46 marker (**Figure 3C**), although in this case the lack of concordance in NKp46 expression between fresh and frozen NK cells was the consequence of a severe reduction of staining upon biobanking with a slope value significantly higher than one (p = 0.0324, F-test), indicating decreased staining after the thawing process (p = 0.0015, Wilcoxon paired test, **Supplementary Figure 1B**, lower panel). Only the percentage of CD57^+^ NK cells showed a highly significant correlation (r = 0.909, p< 0.001, Spearman test) (**Figure 3D**), with a slope value in linear regression analysis close to one (p = 0.3587, F-test). However, significantly lower values were found in frozen PBMC samples (p = 0.0129, Wilcoxon paired test, **Supplementary Figure 1B**, lower panel). This suggests that three of the four NK cell markers assayed were affected by sample biobanking, and so their expression cannot be extrapolated to *in vivo* values. Thus, the validation of the differences of ME/CFS NK subpopulations in our previous study with biobanked PBMCs is discouraged. Although the lack of differences for the highly correlated CD57 marker between frozen PBMCs from individuals with ME/CFS and healthy controls agreed with the findings of our 2013 study, as in the case of T cell markers, no statistical differences for the previously identified ME/CFS NKp46 upregulated markers could be confirmed in frozen PBMCs (**Supplementary Figure 2**). The lack of correlation between NKp46 values in fresh and frozen ME/CFS samples (**Figure 3C**) further argues against future attempts to validate markers in samples other than fresh blood.

### Assessment of New T Cell Immune Markers

**Figure 4:**
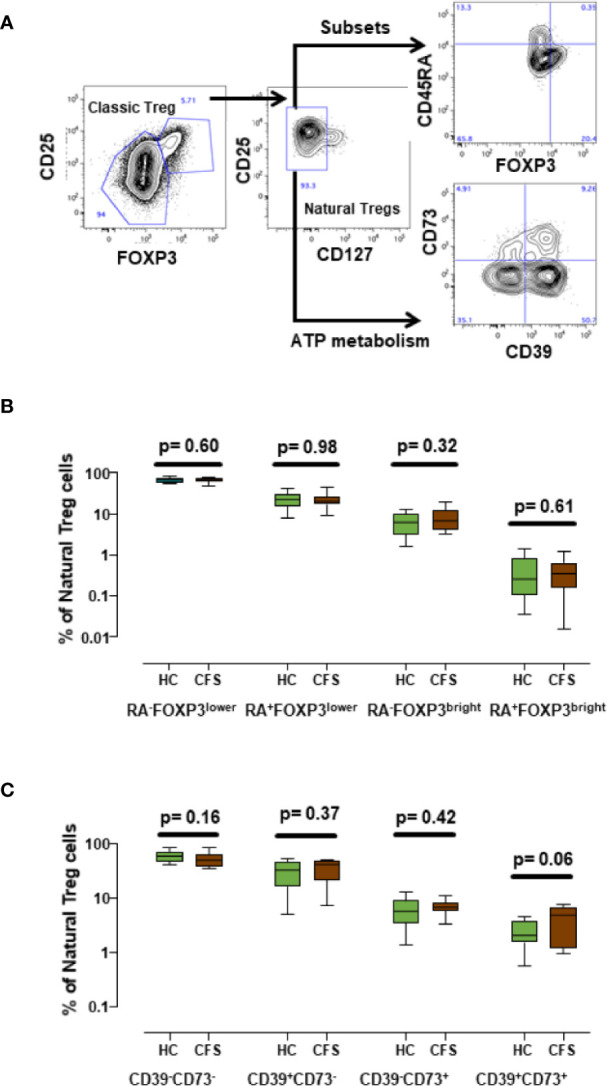
Analysis of new Treg subset markers. **(A)** Gating strategy of Treg cells, first identified by high CD25^+^ and FOXP3^+^ expression (classic Treg) and defined as natural Tregs by the lack of CD127 expression. Coexpression of CD45RA, FOXP3 or co-expression of CD39 and CD73 as new potential Treg markers were assessed as indicated. **(B, C)** Show the frequencies of the indicated cell subsets obtained for healthy controls (n = 18; green) and ME/CFS patients (n = 18; brown). Data are presented as median with interquartile range (boxes) plus minimal and maximal values (bars). *P*-values are indicated for each set of groups compared.

In addition to the immune markers described in our previous study (7), some authors have reported altered expression of other immune markers in ME/CFS patients (9, 17–19), some of them related to Treg cells. Since the Treg compartment is one of the most altered subsets in ME/CFS, we decided to include additional markers which may broaden ME/CFS biomarker validation options with biobanked samples. In particular, we analyzed CD45RA expression to define subpopulations of naïve (CD45RA^+^) and induced/memory (CD45RA^-^) among natural Treg (CD25^+^CD127^-^FOXP3^+^) cells (20). We also identified FOXP3^bright^CD45RA^-^ cell subsets (**Figure 4A**) which were previously reported to correspond to highly active Tregs (21) and analyzed the expression of CD39 (ecto-nucleoside triphosphate diphosphohydrolase-1) and CD73 (ecto-5′-nucleotidase), since these markers play a major role in ATP catabolism and adenosine production (22). Both enzymes are important mediators of Treg activity (23) and have been associated with ME/CFS (24). Our data show that these markers can be readily quantified in frozen cells (**Figure 4A**). However, the comparison of the different Treg cell subsets defined either by CD45RA and FOXP3 or by CD39 and CD73 surface markers did not show statistically significant differences between ME/CFS individuals and healthy controls (**Figures 4B**, **4C**). Interestingly, despite the reduced number of samples analyzed, a trend towards increased levels of CD39^+^CD73^+^ Treg cells in ME/CFS cases compared with healthy controls was observed (p = 0.06, **Figure 4C**), a finding that identifies this new subset of Treg cells, which are also relevant in other chronic pathologies (25) as possible candidate markers that should be explored further in biobanked specimens.

### Intracellular Cytokine Analysis in Frozen Samples

**Figure 5:**
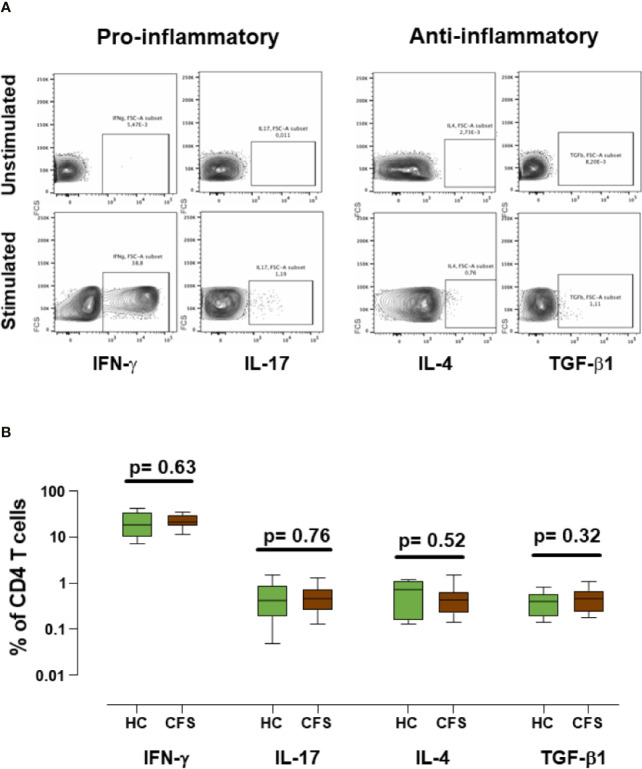
Analysis of cytokine expression in frozen/thawed stimulated samples. **(A)** Representative example of cytokine production from ME/CFS PBMCs by gated CD4^+^ T cells. IFN-γ and IL-17 pro-inflammatory cytokines, and IL-4 and TFG-β1 anti-inflammatory cytokines were evaluated as indicated. **(B)** Percentages of CD4+ cytokine producing T cells are shown for healthy controls (n = 18; green) and ME/CFS patients (n = 18; brown). Data are shown as median with interquartile range (boxes) and minimal and maximal values (bars). Unstimulated conditions were included as negative controls in each experiment. *P*-values are indicated for each set compared.

As changes were found in immune markers associated with the process of cryopreservation of ME/CFS PBMCs, we were interested in testing whether biobanking also affected their cell function. Besides Treg frequency or function, altered levels of circulating inflammatory cytokines and a skewed Th1/Th2 balance have been associated with ME/CFS in recent years (26–29). Therefore, we evaluated the ability of thawed T cells to produce several relevant cytokines. With this aim in mind, cultured T cells were stimulated “*in vitro”* to produce classical pro-inflammatory (IFN-γ and IL-17) and anti-inflammatory cytokines (IL-4 and TGF-β1). TGF-β1 was of particular interest due to its relevance in ME/CFS and in the generation and function of Treg cells (30). Our data show that stimulating cytokine expression from frozen ME/CFS and healthy controls PBMCs is feasible (**Figure 5A**); however, the comparison of cytokine production by stimulated T cells did not show statistically significant differences between cases and healthy controls for any of the cytokines assayed (**Figure 5B**). The observation that T cells could respond to the stimulus applied supports the potential use of biobanked samples for further functional studies of cytokine profiling in ME/CFS. Unfortunately, no data from the corresponding fresh samples were available and therefore the question of whether biobanking affects ME/CFS T cell response to stimulation and cytokine production in different ways remains unanswered.

### Analysis of New Immune Markers in NK Cells in Frozen Samples

**Figure 6:**
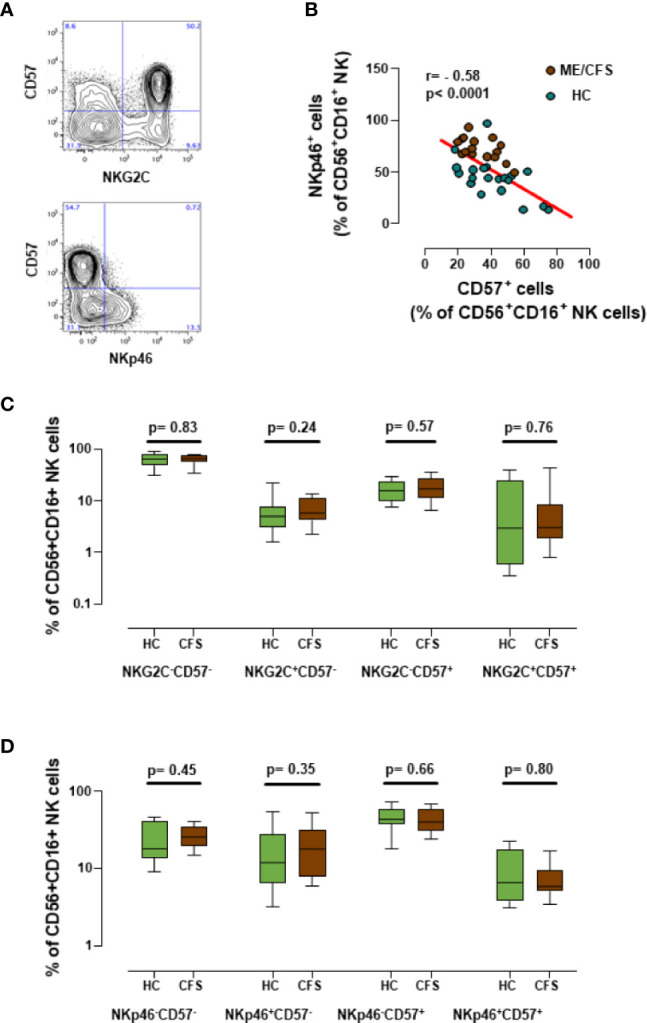
Analysis of new NK cell markers. **(A)** Representative dot-plots for the co-expression of CD57 and either NKG2C or NKp46 in gated CD56^+^CD16^+^NK cells. **(B)** Correlation between CD57 and NKp46 expression in CD56^+^CD16^+^ NK cells from frozen PBMC samples (18 healthy controls in green and 18 ME/CFS patients in brown). Linear regression of data is illustrated by a red line. Spearman correlation coefficients and P-values are shown. **(C, D)** Marker coexpression for healthy controls (n = 18; green) and individuals with ME/CFS (n = 18; brown) are shown as indicated. Data are shown as median with interquartile ranges (boxes) plus minimal and maximal values (bars). *P*-values are indicated for each set compared.

Since over 60% of the ME/CFS cohort reported an infectious trigger (**Table 1**), we expanded the previous characterization of NK cells in ME/CFS cases and healthy controls by analyzing the potential biomarker value of the CD57 expression with NKp46 or NKG2C. The latter is considered an NK-cell surface marker that is strongly affected during infection with herpesvirus and/or cytomegalovirus (31). Although our previous study did not find significant differences in the number of CD57^+^ NK cells between ME/CFS cases and healthy controls, we were still interested in evaluating the CD57 marker further, based on the observation that CD57 MFIs (mean fluorescence intensity) yielded significant differences between groups (7), perhaps indicating the presence of other as yet unidentified NK cell subsets in ME/CFS (i.e., lack of CD94/NKG2C receptor expression). Interestingly, NK cells expressing CD57 and NKG2C or CD57 and NKp46 were detected on thawed cells (**Figure 6A**). Additionally, a clear negative correlation of the latter set of markers (CD57 and NKp46) was evidenced (r = -0.58; p< 0.0001) (**Figure 6B**), suggesting that they are expressed by different NK cell subsets. Although this observation may link increased NKp46 expression to lower CD57 levels in ME/CFS, the comparison of different subsets of NK cell markers defined by NKG2C and NKp46 with CD57 did not show significant differences between cases and controls (**Figures 6C, D**). The observed effect of PMBC freeze/thawing on NKp46 expression (**Figure 3C**) might have limited the discrimination potential of this previously identified ME/CFS marker, either when analyzed alone or in combination with the CD57.

## Discussion

Among the different immunological markers previously shown to be useful for discriminating between ME/CFS and healthy individuals (7), at least four showed notable stability in frozen cells compared to fresh cell samples. These stable markers were: CD57 in NK cells, CD8^+^ effector T cells, CD56 expression in T cells, and Treg frequency. In contrast, other markers showed inconsistent expression in biobanked PBMCs when compared to their fresh counterparts: namely NKp46, CD69 and CD25 expression on NK cells. The reasons for the lack of stability may differ in each case. Indeed, cell surface markers show varying sensitivity to cryopreservation, with some markers, such as CD62L, clearly decreasing due to protein shedding (31). Main lineage markers may also be susceptible to cryopreservation, mainly for NK cells (32, 33) however, the analysis of cryopreserved PBMCs has been standardized for several diseases, in particular HIV infection (34, 35). In contrast, less information is available on other markers, in particular those with a low expression, which may increase their signal due to nonspecific binding to damaged cells. Our data show a clear decrease in NKp46 expression on frozen cells, suggesting a certain level of instability of this molecule on the surface of NK cells that can be explained by its already reported susceptibility to cleavage by metalloproteases during NK cell activation (31). This instability shows that distinguishing between ME/CFS and healthy individuals using biobanked PBMCs is a particularly challenging endeavor; since NKp46 was among the best potential candidate biomarkers identified in fresh blood samples (7), it was one of the main focuses in the present study. Thus, it seems that this major NK-cell activating receptor cannot be validated as a biomarker of ME/CFS using biobanked samples. In addition, the validation of CD25 and CD69 expression in NK cells as potential markers in frozen cells was also clearly compromised by the inconsistent and highly variable levels of expression observed in the biobanked cells, which effectively ruled out their use in future ME/CFS studies with frozen samples.

Although some authors report robust stability among markers, including CD3, CD4, CD8, CD45RO, CD16, CD19, and CD56 after PBMC freeze/thawing (32), others stress the particular sensitivity of T cells (33, 34). Differences in freezing protocols, such as snap-freezing on liquid nitrogen versus the use of isopropyl-alcohol containers and slow pre-freeze at -80°C, or differences in the amounts of DMSO in the freezing medium are known to impact marker cryostability and cell function (32–34). Some authors report that even the time-lapse between cell thawing and surface marker assays by cytometry can determine marker level outcomes and hamper biomarker discovery (33). Even anticoagulant use may impact downstream features of cryopreserved blood cells (32–35). Although the sensitivity to cryopreservation and freeze/thaw under identical conditions might be expected to impact PBMCs from any individual to the same extent, this is in fact not the case: the individual disease state itself may in principle also influence lymphocyte cryosensitivity, particularly in diseases related to immunometabolic imbalance such as ME/CFS. This is an area that deserves future evaluation.

We also analyzed several other parameters such as Treg cell phenotype (naïve, memory and effector, defined by CD45RA and FOXP3 expression levels), Treg functionality assessed by the expression of CD39 and CD73, CD4^+^ T cell cytokine expression profile and the combined expression of CD57 and NKp46 or NKG2C receptors in NK cells. Some of these parameters have previously been explored in ME/CFS patients, with inconsistent findings (6, 18, 24, 36). Our data indicated that the values obtained in frozen cells are similar to those described in the literature for fresh cells (31, 37). However, none of the parameters analyzed showed significant differences between ME/CFS cases and healthy controls. This is of particular relevance in the case of Th17 cells, which represent a key pro-inflammatory subset (38) with functional links to the gut microbiome (39, 40), a field of particular interest in ME/CFS that has been the subject of intense research over the past few years (41, 42). Similarly, no differences in pro- or anti-inflammatory cytokines between ME/CFS and control individuals were noticed in this study. Nevertheless, the low number of PBMC samples included in the present study and the experimental approach (analyzing cytokine-producing cells instead of functional cytokine release) may have hampered the detection of differences between ME/CFS and healthy controls. Exploring other specific (microbial) stimuli, particularly acting on Pattern Recognition Receptors (such as PAMS, DAMPS and TLRs), could be a new strategy for detecting immune dysfunction in ME/CFS.

In conclusion, this study was unable to validate immune biomarkers in ME/CFS, either alone or in combination with other previously defined markers (such as the tandem NKp46 and CD57), which unfortunately showed a high degree of instability in frozen PBMC samples. These data are in agreement with a recent report of a large cohort of frozen PBMC samples from individuals with ME/CFS, and multiple sclerosis and healthy individuals. In that study, similar levels of CD57, NKG2C, and NKp46 expression in NK cells were described in each group; no differences in cytokine profile expression by T cells (IL-2 and IFN-γ) or NK cells (not assessed in our study) were found (18). In contrast, recent reports highlight the relevance of different metabolic determinants or immune markers in ME/CFS (43, 44), suggesting that functional analysis may provide further relevant diagnostic tools, although those assays may require less standardized techniques than flow cytometry. Alternatively, immunometabolic dysfunction may affect cell recovery from ME/CFS frozen PBMC samples (44); however, no differences in cell viability after thawing were observed in our cohort.

The immunophenotypic analysis of frozen samples is a useful tool to characterize immune responses against pathogens and has been used in numerous large intervention multisite studies for a range of medical conditions. However, this kind of approach has limitations, mainly due to the fact that some markers are not stable after the freeze/thawing process of biosamples. For this reason, we believed that it was essential to evaluate the behavior of the immune biomarkers identified for ME/CFS (7) before proceeding with validation studies that would require large numbers of samples and would therefore benefit greatly from the use of biobanked samples. Our data suggest that it is impossible (or at best extremely difficult) to accurately measure certain markers in frozen immune cells, especially CD25 and CD69 in NK cells, but also NKp46, whose expression was strongly reduced after biobanking. The use of metalloprotease inhibitors (45) or specific freezing protocols to protect NK cells (46) may reverse this loss of signal and increase the potential of NKp46 as a biomarker for ME/CFS.

To the best of our knowledge, this is the first study to allow a direct comparison of PBMC immune markers in frozen and fresh ME/CFS samples. It has served to identify important limitations of PBMC storage for use as potential ME/CFS immune markers. However, the small number of frozen samples used may have limited the statistical power to detect differences between groups. In addition, only limited information was available on serological testing, which might have served to identify potential confounding factors. Further investigations are required to clarify more precisely the immune pathomechanisms in people with ME/CFS. Research activity in this area is clearly increasing; with particularly promising data on immunometabolism of “exhausted” phenotype and function, and integrated multi-omics signatures in people with ME/CFS (10, 18, 47).

## Conclusions and Future Directions

In summary, our study suggests for the first time that PBMC sample biobanking impacts some of the previously defined immune markers which may contribute to the diagnosis and prognosis of ME/CFS. The findings indicate the loss of specific PBMC subpopulations or their markers, particularly on T and NK cells. These observations draw attention to the challenges facing immune biomarker discovery in ME/CFS, as they limit the use of frozen specimens from ME/CFS biobanks worldwide. This potentially critical limitation of sample storage should be considered in the design of future studies aimed at exploiting the vast potential of ME/CFS biobanks for exploring large populations of ME/CFS and for further validating new candidate immune biomarkers of the disease.

## Data Availability Statement

The raw data supporting the conclusions of this article will be made available by the authors, without undue reservation.

## Ethics Statement

All subjects provided written signed informed consent prior to participation in accordance with the Declaration of Helsinki. The study protocol was approved by the Health Research Ethics Board of IrsiCaixa, IGTP Committee (reference number: EO-10-007).

## Author Contributions

EG-M, JC, CC, and JB initiated the concept of the project, supervised sampling, and freezing/thawing processing. EO, JC-M, and JB collected and interpreted the data and wrote the manuscript. EG-M, VU, JR, and JA contributed to the study design, supervised the laboratory experiments, analyzed and performed the statistical analysis of data, and also contributed to writing the manuscript. All authors contributed to the article and approved the submitted version.

## Funding

This study was partially supported by the Association of Healthcare Workers for ME/CFS Research (ASSSEM) in Catalonia, Spain. Funders had no role in the study design, data collection and analysis, interpretation, decision of publish, or writing of the manuscript.

## Conflict of Interest

JR is an employee at the Rigau Private Clinic. JB is the founder and CEO and JC is also co-founder and CSO (AlbaJuna Therapeutics, Barcelona, Spain).

The remaining authors declare that the research was conducted in the absence of any commercial or financial relationships that could be construed as a potential conflict of interest.

## Supplementary Material

Supplementary Figure 1 Frequency of different T cell subsets (panel **A**) and NK cell subsets (panel **B**) was assesed in fresh and frozen PBMC samples from individuals with ME/CFS (n = 18). Data are shown as individuals values with means (bars). P-values are shown for Wilcoxon paired test when comparing fresh and frozen samples for each immune marker.

Supplementary Figure 2 Frequency of NK cell subsets was assessed in frozen PBMC samples from 18 healthy controls (HC, green) and 18 ME/CFS patients (CFS, brown) as indicated. Data are displayed as median percentage with interquartile range (boxes), plus minimal and maximal observations (bars). P-values using the Mann-Whitney test are shown for each set compared.
