# Supplementary figures and images for: Impact of Long-Term Cryopreservation on Blood Immune Cell Markers in Myalgic Encephalomyelitis/Chronic Fatigue Syndrome: Implications for Biomarker Discovery
Source: Front Immunol. 2020 Nov 17;11:582330. doi: 10.3389/fimmu.2020.582330 (PMC7732598; doi:10.3389/fimmu.2020.582330)

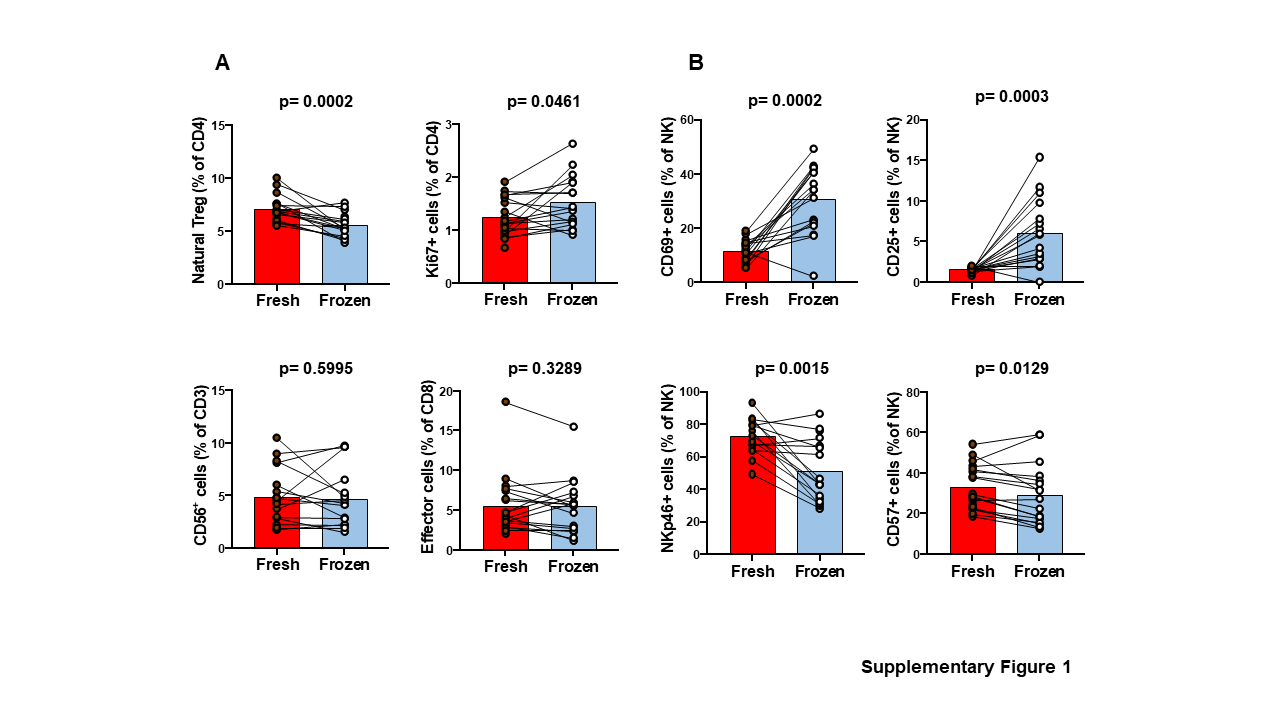

Supplement: Supplementary Figure 1 — Frequency of different T cell subsets (panel A) and NK cell subsets (panel B) was assesed in fresh and frozen PBMC samples from individuals with ME/CFS (n = 18). Data are shown as individuals values with means (bars). P-values are shown for Wilcoxon paired test when comparing fresh and frozen samples for each immune marker. [file Image_1.tif]

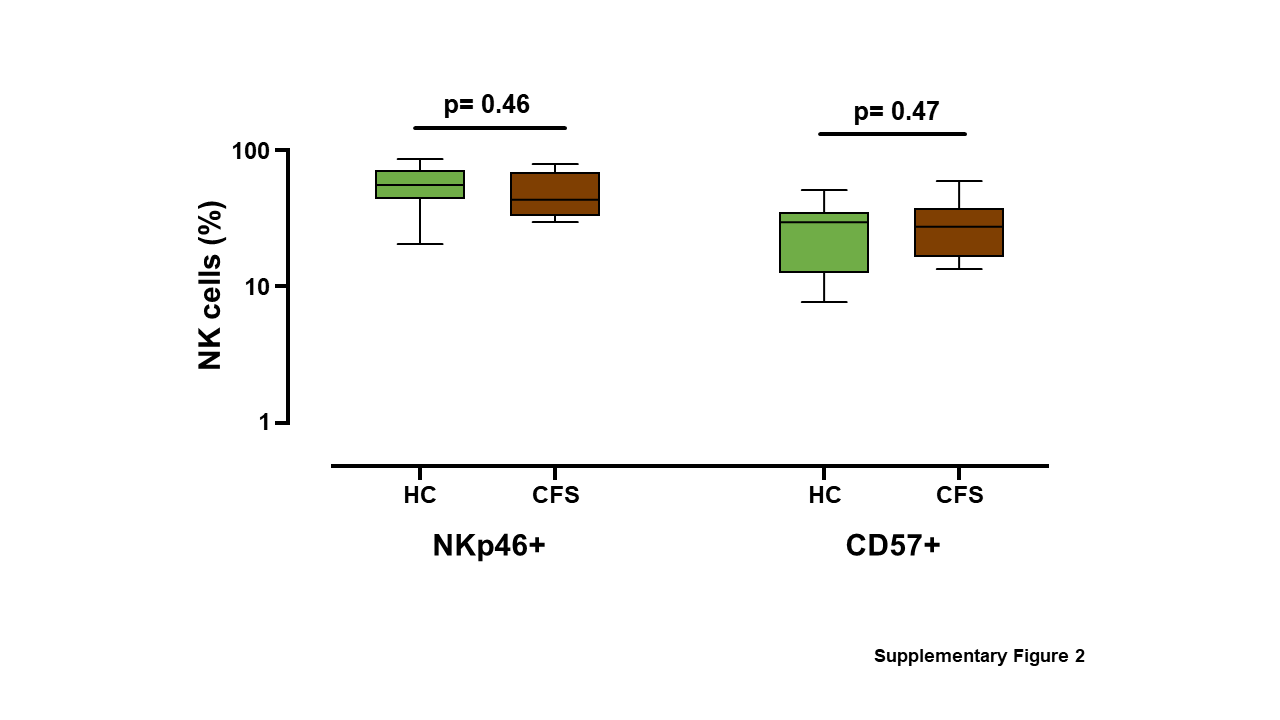

Supplement: Supplementary Figure 2 — Frequency of NK cell subsets was assessed in frozen PBMC samples from 18 healthy controls (HC, green) and 18 ME/CFS patients (CFS, brown) as indicated. Data are displayed as median percentage with interquartile range (boxes), plus minimal and maximal observations (bars). P-values using the Mann-Whitney test are shown for each set compared. [file Image_2.tif]
